# Comparing the Affinity of GTPase-binding Proteins using Competition Assays

**DOI:** 10.3791/53254

**Published:** 2015-10-08

**Authors:** Rosalind C. Williamson, Mark D. Bass

**Affiliations:** ^1^School of Biochemistry, University of Bristol; ^2^Department of Biomedical Science, University of Sheffield

**Keywords:** Molecular Biology, Issue 104, GTPase, Rac1, competition binding, nucleotide loading, cell signaling, Rho-family

## Abstract

In this protocol we demonstrate a method for comparing the competition between GTPase-binding proteins. Such an approach is important for determining the binding capabilities of GTPases for two reasons: The fact that all interactions involve the same face of the GTPases means that binding events must be considered in the context of competitors, and the fact that the bound nucleotide must also be controlled means that conventional approaches such as immunoprecipitation are unsuitable for GTPase biochemistry. The assay relies on the use of purified proteins. Purified Rac1 immobilized on beads is used as the bait protein, and can be loaded with GDP, a non-hydrolyzable version of GTP or left nucleotide free, so that the signaling stage to be investigated can be controlled. The binding proteins to be investigated are purified from mammalian cells, to allow correct folding, by means of a GFP tag. Use of the same tag on both proteins is important because not only does it allow rapid purification and elution, but also allows detection of both competitors with the same antibody during elution. This means that the relative amounts of the two bound proteins can be determined accurately.

**Figure:**
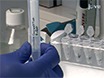


## Introduction

The actin cytoskeleton that determines the shape, polarity and migratory properties of mammalian cells is regulated by the Rho-family of small GTPases. The Rho-family GTPases include RhoA that stimulates cytoskeletal contraction, Rac1 that stimulates actin branching and membrane protrusion, and Cdc42 that has similar effects on actin polymerization to Rac1 and causes the formation of filopodia ^1,2^. GTPase signaling activity is determined by binding of a nucleotide, which controls the contraction and relaxation of the switch I and switch II loops that mediate the protein-protein interactions with both regulators and effectors. Guanosine 5’-triphosphate (GTP)-bound GTPases activate downstream effectors, whereas the Guanosine 5’-diphosphate (GDP)-bound form is inactive. In the cell, cycles of GTP hydrolysis and nucleotide exchange allow rapid turnover of GTPase signals that are necessary for cytoskeletal dynamics. Nucleotide turnover is regulated by three mechanisms. Guanine nucleotide exchange factors (GEFs) stabilize the nucleotide-free GTPase, catalyzing exchange of GDP for GTP, and thereby stimulating GTPase signaling activity ^3,4^. GTPase-activating proteins (GAPs) catalyze hydrolysis of GTP to GDP, thereby inhibiting GTPase signaling activity ^5^. Sequestering molecules such as regulator of chromatin condensation 2 (RCC2) and guanine nucleotide dissociation inhibitors (GDIs) obscure the switch loops and in the case of GDIs remove the GTPase from the membrane by interaction with the prenyl tail ^6,7^. Each of the three classes of regulatory molecule interact with the switch loops, as do the downstream effectors and some trafficking regulators such as coronin-1C ^7^. The purpose of this protocol is to measure competition for the switch I/II binding site between putative regulators and downstream signaling molecules. It should be noted that competition assays test binding to a shared binding site, so that this protocol is not suitable for testing interactions with other sites, such as binding of GDIs to the prenyl tail.

The subtlety of the conformation differences between active and inactive forms, combined with the labile nature of the bound nucleotide, has made study of GTPase-binding events difficult. The role of the bound nucleotide means that conventional binding assays such as immunoprecipitation or surface plasmon resonance are not well suited to investigation, as the nucleotide cannot be controlled. This obstacle is compounded by the overlap in the binding sites of GEFs, GAPs, effectors, sequestering molecules and trafficking molecules, which make binding data for a single interaction difficult to interpret in the context of the competition that will occur in the cell. Immunoprecipitation, in particular, is compromised by competition between binding partners, as under certain cellular conditions, one binding partner might be identified at the expense of all others, while under other conditions, another partner might dominate. The dynamic nature of GTPase signaling is essential to GTPase function and must be considered when analyzing the relationships between the binding interactions of different regulators. Indeed, we recently described a pathway that relied heavily on competitive binding. We identified coronin-1C as a trafficking molecule that bound to the switch loops of GDP-Rac1 ^7^. In areas of low GEF activity, trafficking would dominate, removing Rac1 from those regions. However, when Rac1 is delivered to regions of the cell where GEF activity is high, the GEF would outcompete coronin-1C, thereby both activating Rac1 and preventing coronin-1C-mediated removal of Rac1 from that area. The model goes further, because the action of the GEF exchanges bound GDP for GTP, shifting the equilibrium still further from coronin-1C. Consequently, Rac1 activity could be explained entirely in terms of competition and relative affinity.

In this protocol, we describe a method for comparing the relative affinities of different binding partners for small GTPases, using Rac1 as an example. By using a purified protein approach, it is possible to piece together a chain of signaling events by pair wise comparison, in an experiment where the bound nucleotide can be closely controlled.

## Protocol

### 1. Purification of GST-tagged GTPase

1. Culture an *E. coli* strain such as BL21 transformed with pGEX-Rac1 O/N at 37 °C, shaking at 220 rpm, in 500 ml of autoinduction media (25 mM Na_2_HPO_4_, 25 mM KH_2_PO_4_, 50 mM NH_4_Cl, 5 mM Na_2_SO_4_, 2 mM MgSO_4_, 2 mM CaCl_2_, 0.5% Glycerol, 0.05% Glucose, 0.2% Lactose, 5 g Tryptone, 2.5 g Yeast Extract, 100 µg/ml Ampicillin).
2. Harvest bacteria by centrifugation for 10 min at 10,000 x g, 4 °C.
3. Resuspend bacterial pellet in 20 ml protein extraction reagent, 1x protease inhibitor and incubate for 20 min at RT with inversion.
4. Clarify the lysate by centrifugation at 40,000 x g for 30 min.
5. Add 2 ml glutathione magnetic beads, washed with phosphate-buffered saline (PBS: 10 mM Na_2_HPO_4_, 1.8 mM KH_2_PO_4_, 137 mM NaCl, 2.7 mM KCl).
6. Incubate for 2 hr, mixing by inversion at 4 °C.
7. Wash protein-loaded beads four times with 10 ml PBS, using a magnetic particle sorter to precipitate the beads at each step.
8. Resuspend protein-loaded beads in 2 ml PBS and store at -80 °C in 100 µl aliquots until needed.

### 2. Expression of GTPase-binding proteins

1. The day before the experiment, transfect plasmids encoding green fluorescent protein (GFP)-tagged versions of each GTPase-binding protein into a separate 75-cm^2^ flask of HEK293T as follows. For validation of nucleotide loading, transfect GFP-tagged TrioD1 into a third 75-cm^2^ flask of HEK293T.
  1. Dilute polyethylamine to 1 mg/ml in 100 µl sterile 150 mM NaCl.
  2. Add 27 µl diluted polyethylamine to 223 µl reduced serum media.
  3. Add 12 µg plasmid DNA to 250 µl reduced serum media.
  4. Incubate each tube for 2 min at RT.
  5. Combine the polyethylamine and DNA mixes in a single tube and vortex for 2 min.
  6. Incubate for 15-20 min at RT.
  7. Replace the growth media (Dulbecco’s Modified Eagle Media, 10% fetal bovine serum, 2 mM L-glutamine, no antibiotics) on 90% confluent HEK293T with 5 ml fresh growth media.
  8. Add the combined polyethylamine/DNA mix to the flask and incubate O/N at 37 °C, 5% CO_2_.

### 3. Purification of GTPase-binding proteins

1. Rinse flasks of transfected cells in PBS and drain flask for 5 min, aspirating free liquid.
2. Scrape off cells in 500 µl lysis buffer (50 mM Tris-HCl (pH7.8), 1% Nonidet P-40, 1x protease inhibitor) into microfuge tube.
3. Lyse cells by mixing by inversion at 4 °C for 30 min.
4. During lysis, wash two lots of 40 µl GFP-Trap beads three times with fresh lysis buffer, sedimenting beads at 2,700 x g for 2 min between washes.
5. Clarify lysates by centrifugation at 21,000 x g for 10 min.
6. Transfer clarified lysate of each of the competitor proteins to separate washed GFP-trap beads and allow GFP-fusion proteins to bind for 2 hr, mixing by inversion at 4 °C. Keep lysate from GFP-TrioD1 cells on ice.
7. Wash loaded GFP-Trap beads twice in 50 mM Tris-HCl (pH 7.8), 50 mM NaCl, 0.7% (w/v) Nonidet P-40 and twice in 50 mM Tris-HCl (pH 7.6), 20 mM MgCl_2_, sedimenting beads at 2,700 x g for 2 min between washes.
8. Elute GFP-fusion proteins by adding 40 µl 0.2 M glycine (pH 2.5) and pipetting up and down for 30 sec. Immediately sediment beads at 21,000 x g for 60 s and transfer liquid to a new microfuge tube containing 4 µl 1 M Tris-HCl (pH 10.4). Do this quickly to limit damage to the purified protein.
9. Analyze 1 µl of each purified protein by Western blot and probe with an anti-GFP antibody to establish relative yield using a quantitative blotting system according to manufacturer’s protocol. Alternatively, determine protein concentrations by bicinchoninic acid (BCA) assay but this introduces errors if the proteins do not react with the assay in an identical fashion or there are contaminant proteins.
10. Equalize molar protein concentration by the addition of 50 mM Tris-HCl (pH 7.6), 20 mM MgCl_2_.

### 4. Nucleotide loading of GTPase

1. Thaw one aliquot of GST-Rac1 magnetic beads, prepared in step 1.
2. Take 90 µl of GST-Rac1 beads and wash three times with 20 mM Tris-HCl (pH 7.6), 25 mM NaCl, 0.1 mM DTT, 4 mM EDTA, using a magnetic particle sorter to precipitate the beads at each step.
3. Aspirate buffer from beads and add 100 µl 20 mM Tris-HCl (pH 7.6), 25 mM NaCl, 0.1 mM DTT, 4 mM EDTA.
4. According to whether GDP, GTP or no nucleotide loading is required for the competition experiment, add 12 µl 100 mM GDP, 12 µl 10 mM guanosine 5’-[γ-thio]triphosphate (GTPγS) or no nucleotide to 60 µl GST-Rac1 beads.
5. For the nucleotide-loading controls, split the remaining beads into three 10-µl aliquots and add 2 µl 100 mM GDP, 2 µl 10 mM GTPγS or no nucleotide to each tube.
6. Incubate bead mixes for 30 min at 30 °C with agitation.
7. Stabilize nucleotide-bound Rac1 by addition of 1 M MgCl_2_: 3 µl to the experimental mix (step 4.4), 0.5 µl to each of the control mixes (step 4.5).

### 5. Competition binding.

1. Set up 6 microfuge tubes, each containing: 200 µl 50 mM Tris-HCl (pH 7.6), 20 mM MgCl_2_ 10 µl experimental nucleotide-loaded Rac1 beads (from step 4.7) 5 µl Rac1-binding protein A (constant binding protein)
2. To each tube, add 0, 1, 2.5, 5, 10 or 20 µl Rac1-binding protein B (variable binding protein). These volumes assume approximately equal stock concentrations of the constant and variable binding proteins and may need to be adjusted.
  1. Adjust volumes of binding proteins A and B if there are large differences in the binding affinities of the two proteins and this should be determined empirically through the experimental repeats. Make up the total volume of the binding mixture to 235 µl by addition of 50 mM Tris-HCl (pH 7.6), 20 mM MgCl_2_.
3. Set up a microfuge tube containing: 200 µl 50 mM Tris-HCl (pH 7.6), 20 mM MgCl_2_ 10 µl experimental nucleotide-loaded Rac1 beads (from step 4.7) 10 µl Rac1-binding protein A (constant binding protein)
4. Set up the GDP, GTPγS and no nucleotide control tubes: 200 µl 50 mM Tris-HCl (pH 7.6), 20 mM MgCl_2_ 10 µl control Rac1 beads loaded in step 4.5 with GDP, GTPγS or no nucleotide and stabilized in step 4.7. 180 µl HEK293T GFP-TrioD1 lysate, prepared as in step 3.6 4 µl 1 M MgCl_2_
5. Incubate the mixture for 2 hr, mixing by inversion at 4 °C.
6. Wash the beads three times with 50 mM Tris-HCl (pH 7.6), 20 mM MgCl_2_.
7. Elute bound proteins in 20 µl reducing sample buffer (50 mM Tris-HCl (pH 7), 5% SDS, 20% glycerol, 0.02 mg/ml bromophenol blue, 5% β-mercaptoethanol).

### 6. Analysis of competition

1. Resolve 10 µl of the bound protein (step 5.6) by sodium dodecyl sulfate polyacrylamide gel electrophoresis (SDS-PAGE) and Western blot.
2. Incubate the membrane at 4 °C O/N in anti-GFP antibody diluted 1/1000 in Blocking Buffer diluted to 1x in PBS, 0.1% Tween-20 to detect both of the tagged GTPase-binding proteins.
3. Wash the membrane three times for 10 min with PBS, 0.1% Tween-20.
4. Incubate the membrane for 30 min at RT in DyLight 800-conjugated anti-rabbit secondary antibody, diluted 1/10,000 in Blocking Buffer diluted to 1x in PBS, 0.1% Tween-20.
5. Wash the membrane three times for 10 min with PBS, 0.1% Tween-20.
6. Scan the membrane using an infrared imaging system, using the software to measure band intensity according to manufacturer’s protocol.
7. Plot the band intensity of each protein against volume of the variable competitor (Protein B).
8. Divide the volume of variable competitor at the point at which the lines intersect by the volume of constant competitor (Protein A, 5 µl) to determine the competitor ratio at which equilibrium is achieved.
9. For validation of nucleotide-loading status, probe membranes for p21-activated kinase 1 (PAK1) (an effector) and GFP-TrioD1 (a GEF), as described in steps 6.1-6.6.

## Representative Results

This protocol is designed to calculate the relative affinities of binding partners for Rac1, without the need to know the precise concentration of the competitors (**Figure 1**). Determination of protein concentration introduces errors and when considering competition between molecules in a signaling pathway is not needed. However, it is important to know that the two competitors have the same molar concentration in the stock solutions to allow simple ratios to be calculated when adding different volumes to the assay. 40 µl of GFP-Trap beads have a binding capacity of ~300 pmol so a confluent 75 cm^2^ flasks of highly expressing cells will saturate the beads, with the result that the preparations of the two different binding proteins will be similar before adjustment (**Figure 2A**). If one of the proteins expresses poorly, this problem can be overcome by purifying that protein from more than one flask of cells.

The binding of most GTPase effectors and regulators depends on the nucleotide-loading of the bait GTPase, so it is important to test whether loading has been successful. Loading can be verified by precipitating known binding proteins from cell lysates. Effector proteins, such as PAK1 bind to GTP-Rac1 and can be easily precipitated from lysates and detected by Western blotting ^8^ (**Figure 2B**). GEFs bind preferentially to nucleotide-free GTPase to stabilize the transition state. As GEFs are of low abundance, usually inactive and frequently blot poorly, it is better to overexpress a GEF or GEF fragment to test nucleotide-free GTPase. We frequently use the first Dbl homology of Trio, expressed as a GFP fusion (GFP-TrioD1 ^9^) (**Figure 2B**) but any GEF would work. Proteins that bind to the GDP-loaded GTPase are rarer. We recently reported RCC2 as one such protein ^7^, or GDP-loading can be validated simply as binding to neither GEF nor effector.

The output from the experiment will be a Western blot depicting the two GFP-tagged binding partners bound to the GTPase. By using a single antibody to detect both proteins, the concentrations at which similar amounts of both competitors bind can be determined and therefore the relative affinities inferred. In this example competition between the propeller domain of the Rac1-trafficking protein, coronin-1C (Rac1-binding protein A), and the Rac1-sequestering protein, RCC2 (Rac1-binding protein B), is demonstrated (**Figure 3A**). By using a constant volume of coronin-1C propeller (5 µl), and adding increasing volumes of RCC2, we can see from the GFP blot that equilibrium is reached at 1.25-2.5 µl of RCC2 (asterisk), demonstrating that RCC2 has a stronger affinity for Rac1 than coronin-1C. By measuring the intensity of bands using quantitative Western blotting, and plotting average values for each competitor, the equilibrium point can be calculated accurately by identifying the volumes at which the curves intersect (**Figure 3B**).

One of the possible obstacles to a successful competition assay is if the binding partners bind to one another as well as binding to Rac1. In **Figure 3A+B** we demonstrate competition between RCC2 and the propeller domain of coronin-1C, rather than full-length coronin-1C. The reason for using the truncated coronin is that coronin-1C also binds RCC2 through the tail domain. When full-length coronin-1C is titrated against RCC2, binding of both proteins is detected, due to ternary complex formation, rather than competition (**Figure 3C**). If competition is occurring, binding of one protein will increase while the other decreases, and total bound GFP-fusion will remain constant. In cases where a ternary complex forms it is necessary to truncate one of the GTPase-binding protein so that the competitors no longer interact.


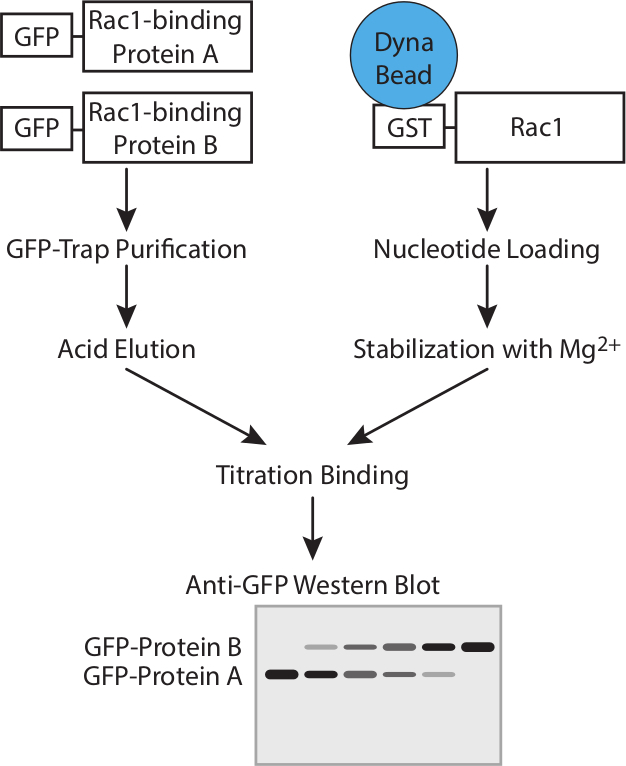
**Figure 1. Workflow. **Schematic representation of the workflow for determining the affinity of GTPase-binding proteins using competition assays. Please click here to view a larger version of this figure.


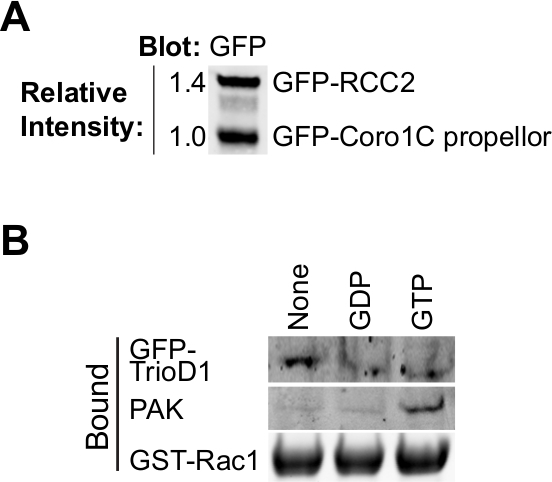
**Figure 2. Validation of purified proteins. (A) **Purified GFP-tagged Rac1 binding proteins analyzed by Western blot, probing with anti-GFP to determine the relative yield of the two proteins. This type of equalization during the experiment allows the concentration of the two proteins to be adjusted so they match in the binding experiment. **(B) **GDP, GTPγS and no nucleotide-loaded GST-Rac1 was incubated with lysate from HEK293T expressing GFP-TrioD1 and bound proteins detected by plotting for endogenous PAK1 or overexpressed GFP-TrioD1. Please click here to view a larger version of this figure.


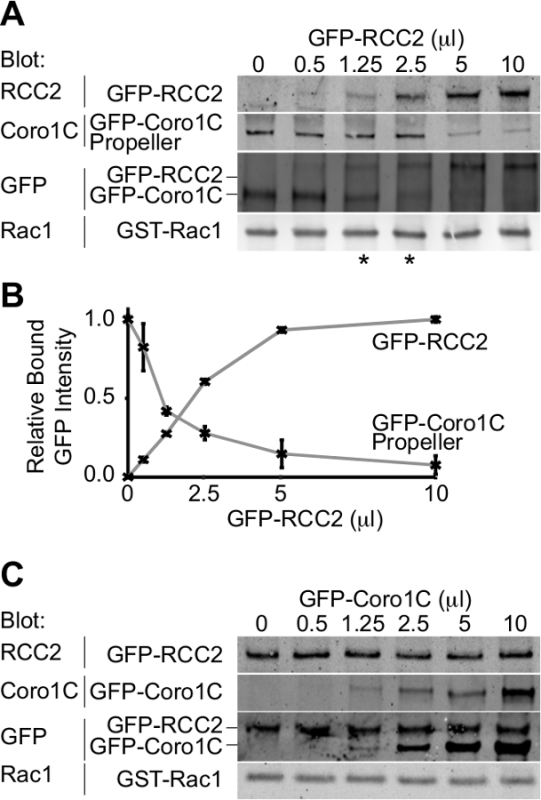
**Figure 3. Western blot analysis of relative protein binding.** Example outputs from competition-binding assays. **(A)** GDP-loaded Rac1 was mixed with 5 µl GFP-coronin-1C propeller domain and increasing volumes of GFP-RCC2 were titrated in. By Western blotting bound proteins for GFP, issues with differential detection of the two proteins are avoided and the GFP signal reports the molar ratio between the two fusion proteins. Asterisks indicate the competition ratios on either side of the equilibrium point. **(B)** Band intensities of bound GFP fusion proteins from three independent experiments were measured by quantitative Western blotting, using fluorophore-conjugated secondary antibodies and averages plotted to calculate the amount of RCC2 needed to reach equilibrium. **(C)** Example output from an experiment where Rac1-binding proteins bind to one another and form a ternary complex, rather than competing. GDP-loaded Rac1 was mixed with 5 µl GFP-RCC2 and increasing volumes of GFP-coronin-1C full-length were titrated in. The increase in bound GFP-coronin-1C without loss of bound GFP-RCC2 indicates ternary complex formation. Please click here to view a larger version of this figure.

## Discussion

This protocol describes a method for comparing the relative affinities of pairs of small GTPase-binding proteins. The key steps are the preparation of purified GTPase-binding proteins and the nucleotide loading of the GTPase. The use of GTPase-binding proteins with the same GFP tag, allows the concentrations at which similar amounts of each competitor binds to be accurately determined. The use of recombinant nucleotide-loaded GTPase allows interrogation of the binding properties of the GTPase under specific activity conditions. This step is also the most sensitive as nucleotides will both hydrolyze and detach from the GTPase if the magnesium conditions are not maintained precisely.

In the cell, the large number of GTPase-binding proteins combined with the rapid nucleotide turnover makes such pathways difficult to interpret. The simplicity of this method in comparing only pairs of binding proteins and using carefully controlled nucleotide-loading conditions allows signaling pathways to be elucidated. However, the greatest strength of the protocol is also the greatest weakness as it is a simplification of the *in vivo* situation. Competition assays can be used to build a robust hypothesis, but this should then be tested in cells by knockdown experiments.

There are three features that must be considered when selecting the GFP-tagged GTPase-binding proteins to be used in the experiment. First, the fusion proteins must express well in mammalian cells, such as HEK293T, as competition assays require a reasonable amount of protein. Second, it must be possible to purify the recombinant protein without significant degradation, and where this is not possible, cloning of a GTPase-binding fragment should be considered. Third, the two GTPase-binding proteins must resolve from one another on SDS-PAGE to allow analysis in section 6.

There are a number of potential caveats to the experiment that need to be considered, and possibly addressed:

Possible denaturation of purified GTPase-binding proteins during the acid elution step or steric hindrance by the GFP tag. In our hands, these have not been a problem, but must be tested. The purified proteins can be tested in functional assays ^10^. Commercial kits now exist for testing the activity of GEFs or GAPs without the need for isotope-labeled nucleotides. Sequestering proteins, by their nature protect GTPases from GEF or GAP activity, so can be used as competitive inhibitors in the commercial GEF or GAP assays, as we did in our recent publication ^7^. The relevant feature of proteins that traffic GTPase are the capacity to bind the GTPase, and this can be tested easily in a pull down assay. An alternative approach to testing protein integrity that is applicable to all binding proteins is to titrate protein eluted from GFP-trap beads with glycine with the same protein removed from GFP-trap beads by enzymatic cleavage. The experiment would be analyzed by probing both the GFP-tagged and cleaved protein with an antibody against the protein itself. If the protein is undamaged by elution, equilibrium should be achieved at a 1:1 ratio. This approach would also indicate whether the presence of the GFP tag itself compromises the binding properties of the candidate protein, though this does require the production of a construct with an enzymatic cleavage site between the tag and the binding protein. Whether the protein is compromised by the tag or the elution step, the problem could be addressed by modifying the protocol to use an alternative purification method. Rather than GFP, binding proteins could be His-tagged, purified using Ni-NTA and analyzed using an antibody against the His-tag. The important feature is that both binding proteins must share a common tag although, if necessary, two tags could be added to a protein, one for purification and the other for detection.

The protocol is designed to investigate competition between interactions with the switch I/II domains. Although the majority of GTPase interactions are mediated by this motif, there are some exceptions, most notably the interactions of GDIs that bind to the prenyl tail, as well as obscuring the switch domains. In principle, the protocol could be adapted to use GTPase purified from mammalian cells, so that the GTPase is prenylated, however, the presence of multiple binding sites or allosteric effects complicate the interpretation of competition-binding data. Further problems associated with such a modification are that GDIs co-purify with GTPase from mammalian cells, compromising the purity of the isolated proteins and the hydrophobic nature of the prenyl groups means that prenylated GTPases are associated with either GDI or lipid membrane and such factors would need to be considered in the experiment.

The amount of GST-Rac1 being used in the assay. The constant GTPase binding protein must be at a greater concentration than the Rac1, or when the competitor is added, it will simply bind to free Rac1. It will be immediately obvious if this has happened as binding of the competitor, without a loss of the constant protein, will be detected in much the same way as when the two competing proteins bind to one another as shown in **Figure 3B**. As an additional control (Step 5.3), a binding reaction containing double the amount of constant binding protein and no variable binding protein should be included (Step 5.3). If the Rac1 in the titration experiment is saturated, doubling the amount of constant binding protein will have no effect on the output. The volumes suggested in the protocol should be appropriate, but the amount of Rac1 can be easily reduced. If binding of the competitor without loss of the constant binding partner is observed, reducing the amount of Rac1 should be attempted before trying to map binding sites to avoid ternary complex formation.

Non-specific interaction of GTPase-binding proteins with the GST or bead, as well as specifically with Rac1. This problem would be manifested by residual binding of the constant GTPase-binding protein, even when the variable GTPase-binding protein has reached a plateau at high concentration. Identification of this issue will be aided by conducting reciprocal experiments where the constant and variable GTPase-binding proteins are swapped. Reciprocal experiments will also greatly improve the accuracy of the estimate of equilibrium point, so should always be included. In cases of non-specific binding, the relative concentrations at which equilibrium is achieved can still be calculated by comparing band intensity between the maxima and minima for each protein, or by measuring the extent of non-specific binding by using GST beads as bait, rather than GST-Rac1.

Pull down assays using different nucleotide-loading conditions should be used to complement the competition assay described in this protocol. Determining the nucleotide preference of partners is important for both understanding the competition events and understanding the signaling pathway that the GTPase-binding protein is involved in. In **Figure 2B** we analyze binding of proteins with established preference for GTP-loaded or nucleotide-free GTPase as a means to validate nucleotide loading. However, it is sensible to investigate the effect of nucleotide loading on each of the competitors as well. If the hypothetical competitors show different preferences, competition will make less of a contribution to the signaling pathway, and indeed nucleotide turnover is likely to be the mechanism that directs exchange of the binding proteins.

## Disclosures

The authors have nothing to disclose.

## References

[B0] Burridge K, Rho Wennerberg K (2004). and Rac take center stage. Cell.

[B1] Raftopoulou M, Hall A (2004). Cell migration: Rho GTPases lead the way. Dev Biol.

[B2] Rossman KL, Der CJ, Sondek J (2005). GEF means go: turning on RHO GTPases with guanine
nucleotide-exchange factors. Nat Rev Mol Cell Biol.

[B3] Worthylake DK, Rossman KL, Crystal Sondek J (2000). structure of Rac1 in complex with the guanine nucleotide exchange
region of Tiam1. Nature.

[B4] Scheffzek K, Ahmadian MR (2005). GTPase activating proteins: structural and functional insights 18
years after discovery. Cell Mol Life Sci.

[B5] Del Pozo, A M (2002). Integrins regulate GTP-Rac localized effector interactions
through dissociation of Rho-GDI. Nat Cell Biol.

[B6] Williamson RC (2014). Coronin-1C and RCC2 guide mesenchymal migration by trafficking
Rac1 and controlling GEF exposure. J Cell Sci.

[B7] Del Pozo MA, Price LS, Alderson NB, Ren XD, Schwartz MA (2000). Adhesion to the extracellular matrix regulates the coupling of
the small GTPase Rac to its effector PAK. Embo J.

[B8] Van Rijssel J, Hoogenboezem M, Wester L, Hordijk PL, Van Buul JD (2012). The N-terminal DH-PH domain of Trio induces cell spreading and
migration by regulating lamellipodia dynamics in a Rac1-dependent
fashion. PLoS.

[B9] Self AJ, Hall A (1995). Measurement of intrinsic nucleotide exchange and GTP hydrolysis
rates. Methods Enzymol.

